# The European Prevention of Alzheimer's Dementia Programme: An Innovative Medicines Initiative-funded partnership to facilitate secondary prevention of Alzheimer's disease dementia

**DOI:** 10.3389/fneur.2022.1051543

**Published:** 2022-11-22

**Authors:** Stina Saunders, Sarah Gregory, Matthew H. S. Clement, Cindy Birck, Serge van der Geyten, Craig W. Ritchie

**Affiliations:** ^1^Edinburgh Dementia Prevention, Centre for Clinical Brain Sciences, University of Edinburgh, Edinburgh, United Kingdom; ^2^Alzheimer's Disease Data Initiative, Kirkland, WA, United States; ^3^Alzheimer Europe, Luxembourg, Luxembourg; ^4^Janssen Research and Development, Division of Janssen Pharmaceutica NV, Beerse, Belgium; ^5^Brain Health Scotland, Edinburgh, United Kingdom

**Keywords:** Alzheimer's disease, Longitudinal Cohort Study, public-private partnership, Innovative Medicines Initiative, secondary prevention

## Abstract

**Introduction:**

Tens of millions of people worldwide will develop Alzheimer's disease (AD), and only by intervening early in the preclinical disease can we make a fundamental difference to the rates of late-stage disease where clinical symptoms and societal burden manifest. However, collectively utilizing data, samples, and knowledge amassed by large-scale projects such as the Innovative Medicines Initiative (IMI)-funded European Prevention of Alzheimer's Dementia (EPAD) program will enable the research community to learn, adapt, and implement change.

**Method:**

In the current article, we define and discuss the substantial assets of the EPAD project for the scientific community, patient population, and industry, describe the EPAD structure with a focus on how the public and private sector interacted and collaborated within the project, reflect how IMI specifically supported the achievements of the above, and conclude with a view for future.

**Results:**

The EPAD project was a €64-million investment to facilitate secondary prevention of AD dementia research. The project recruited over 2,000 research participants into the EPAD longitudinal cohort study (LCS) and included over 400 researchers from 39 partners. The EPAD LCS data and biobank are freely available and easily accessible *via* the Alzheimer's Disease Data Initiative's (ADDI) AD Workbench platform and the University of Edinburgh's Sample Access Committee. The trial delivery network established within the EPAD program is being incorporated into the truly global offering from the Global Alzheimer's Platform (GAP) for trial delivery, and the almost 100 early-career researchers who were part of the EPAD Academy will take forward their experience and learning from EPAD to the next stage of their careers.

**Discussion:**

Through GAP, IMI-Neuronet, and follow-on funding from the Alzheimer's Association for the data and sample access systems, the EPAD assets will be maintained and, as and when sponsors seek a new platform trial to be established, the learnings from EPAD will ensure that this can be developed to be even more successful than this first pan-European attempt.

## Introduction

Early detection of Alzheimer's disease (AD) pathology offers an opportunity for intervention, either to delay symptom onset or to stop the disease development entirely. Due to the long silent period in the AD pathology where the disease starts developing more than 20 years before traditional symptoms of dementia manifest (1, 2), identifying individuals at risk of dementia in pre-dementia stages is a major aim of many disease-modifying therapies currently developed for AD. However, because of the stage of illness that patients present with in current *memory clinics*, clinical trials commonly recruit individuals who are in the more advanced stages of the disease and there is a dearth of knowledge in the longitudinal modeling of AD trajectories in the preclinical period of disease to inform trial design. Moreover, recruitment rates for AD research remain low, resulting in drug studies commonly missing recruitment targets (3). To this end, the European Prevention of Alzheimer's Dementia (EPAD) program was established in 2015, funded by the European Union's Innovative Medicines Initiative (IMI), and is now succeeded by the Innovative Health Initiative.

EPAD aimed to assist in the development of interventions for the secondary prevention of AD. The program set out to develop a clinical trial platform that could test multiple interventions concurrently in a multitude of sites across Europe. Individuals recruited by these sites were highly phenotyped and formed a readiness cohort referred to as the EPAD Longitudinal Cohort Study (LCS). The first participant consented in May 2016, and until the study closure in March 2020, over 2,000 research participants eligible for secondary-prevention studies were recruited into the EPAD LCS, generating several million data points and over 1 million aliquots of cerebrospinal fluid (CSF), plasma, serum, saliva, and urine (4). Due to the longitudinal nature of the study, participants completed a varying number of visits which are detailed in the results section.

EPAD stemmed from a need to develop new pharmacological agents for AD where there had been a significant lack of progress over 15 years at the time. Individuals recruited into the EPAD LCS were aimed to fill the continuum of low to high risk of developing AD but not have dementia. Although the original focus of the EPAD proposal had been on preclinical AD (evidence of AD pathology with no manifest symptoms), the funded EPAD project expanded recruitment to include people with prodromal AD (evidence of AD and manifest symptoms, although insufficient to satisfy criteria for dementia). There were several reasons for why a platform trial design was chosen for EPAD. A platform trial enabled (1) a single operational environment, (2) a single master protocol (including sharing placebo data), (3) a site network and community that conducted all three elements of research participant engagement (register, cohort, and trial), and, therefore, (4) a single sponsor to oversee the whole program under a single governance framework.

In the current article, we summarize the key findings of the EPAD study to date, detail the data access policy, and define the substantial residual assets of the EPAD project for the scientific community, patient population, and industry. Additionally, we describe the EPAD structure with a focus on how the public and private sectors interacted and collaborated within the project, reflect how IMI specifically supported the achievements of the above outputs, and conclude with a view for future.

## Methods

The EPAD program was the winning response to a call put out by IMI to undertake deep phenotyping of individuals at risk of AD to determine their eligibility for a secondary-prevention Proof of Concept (PoC) trial. It was recognized that deep phenotyping would reduce screen failures in PoC (drug trials) as knowing amyloid status, cognitive function, medical comorbidities, and *Apolipoprotein E* (*APOE*) status before invitation to the PoC trial would enable approaching individuals who are already deemed eligible per the PoC study protocol. IMI was uniquely positioned to fund such an innovative platform trial in AD as it brings together the pharmaceutical industry [under the European Federation of the Pharmaceutical Industry and Associations (EFPIA)], academia, the third sector, and small and medium enterprises (SMEs).

### Private-public partnership

EPAD had a budget of €64 million, involved 39 partners which operated across 29 sites in 10 countries in Europe, and (at its peak of activity) had 410 people from Europe and the USA receiving direct salary costs from the grant. As a public-private partnership, the EPAD coordination was shared by partners from academia and EFPIA. The private-public partnership was achieved through all governance entities and work packages being jointly led by an EFPIA and an academic lead.

### Study management

**Figure 1:**
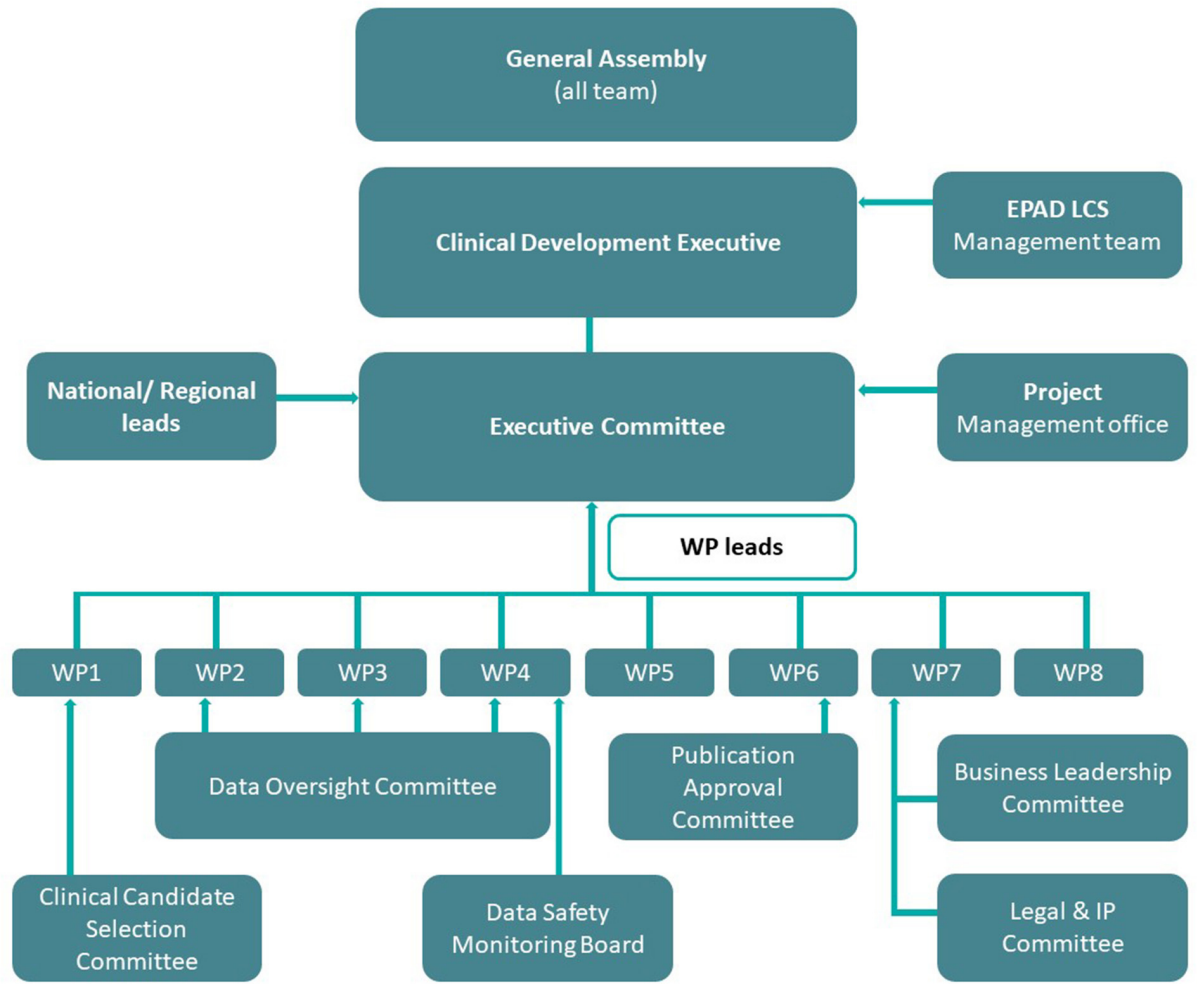
EPAD governance structure. Work Package 1: Scientific Challenges; Work Package 2: Statistical/Methodology Engine Room; Work Package 3: Parent Cohorts and EPAD Register; Work Package 4: EPAD Cohort and EPAD Trials; Work Package 5: Project Management; Work Package 6: Dissemination; Work Package 7: Business Model and Sustainability; Work Package 8: Ethical, Legal and Social Implications.

EPAD was managed by the executive committee which met monthly and had a balanced representation from EFPIA, academia, and the project management office. From an operational perspective, EPAD was divided into eight work packages (WPs; Figure 1) with representatives again from industry and academia (5). These work packages were complimented by transversal working groups and committees that dealt with specific needs at various stages of the program's development. Data support was provided by numerous partners, that is, IXICO (neuroimaging partner), Aridhia (data-management partner), and IQVIA (clinical research organization). Moreover, from the outset, it was recognized that the value of the data collected in the EPAD LCS (and PoC trial) would be at a breadth and scale to help facilitate conceptual advance in the understanding of disease models in the early phases of neurodegeneration. The EPAD data, therefore, had to be both open access and of the highest quality.

### Structure

The EPAD LCS was set up to collect longitudinal data for disease modeling purposes (6) and also to act as a readiness cohort for PoC trials. Although the observational LCS was successfully undertaken throughout Europe, the IMI funding period ended without the PoC trials starting. The objective of the PoC trial was to develop a platform and master protocol for a perpetual, Bayesian adaptive trial for the secondary prevention of AD dementia. To create readiness for a trial to start, EPAD built and certified trial delivery centers (TDCs) across Europe which undertook the cohort study and were approved and highly qualified to conduct PoC trials thereafter.

### Participant involvement

Finally, EPAD also recognized from the outset that all clinical research projects benefit from the insights of people with lived experience, either as research participants and/or those affected by the disease. At a national level, the research participants were coordinated into national panels, who would discuss their experience of the LCS and help design communication materials. These national panels would also be asked to provide formal feedback on protocol amendments. By 2019, four national panels had been established, in Spain, the Netherlands, England, and Scotland, and each panel sent representation to the annual EPAD general assembly (7).

## Results

### Open access data: The EPAD LCS dataset

The most substantial output from the IMI period of EPAD was the EPAD LCS, recruiting 2,096 research participants of whom 1,828 were available for analysis (Table 1). The EPAD LCS dataset is unique, whereby 37% of the sample were amyloid positive at the point of enrollment to the study (CSF Aβ <1,000 pg/ml using the Roche Diagnostic Elecsys_®_ System) (8). This resulted in *n* = 358 deeply phenotyped participants who fill the criteria for preclinical AD. As the LCS finished in Spring 2020, a small proportion of the early recruits completed 3 years of follow-up and four study visits (baseline, month 6, month 12, month 24, and month 36) (Table 2).

**Table 1:** Baseline characteristics of 1,843 non-screen failed participants in the EPAD LCS (8).

**Variable**		**Mean (SD)**	**Frequency (%)**	**Number currently unknown**
Gender	Female		1,035 (56.6%)	
	Male		793 (43.4%)	
Age, years		65.7 (7.41)		
Age group	Under 75 years old		1,612 (88.2%)	
	75 years old and above		216 (11.8%)	
	Years of formal education^*^	14.4 (3.70)		
Education	Up to secondary		722 (39.5%)	
	Beyond secondary to ordinary first degree		451 (24.7%)	
	Postgraduate studies		655 (35.8%)	
Family history of AD?	No		657 (35.9%)	
	Yes		1,171 (64.1%)	
*APOE* ε4 genotype	No *APOE ε4* alleles		1,077 (58.9%)	
	One *APOE ε4* allele		618 (33.8%)	57
	Two *APOE ε4* alleles		76 (4.2%)	

* Years of education is country-specific.

**Table 2:** Number of completed research participant visits and availability of key assessment data at each visit (8).

	**Visit 1**	**Visit 2**	**Visit 3**	**Visit 4**	**Visit 5**
	**(baseline)**	**(6 months)**	**(1 year)**	**(2 years)**	**(3 years)**
*N*	2,096	1,571	1,190	397	90
**Break-down of number of samples per visit**					
Blood samples (*APOE*^a^)	2,007	0	0	0	0
MRIs^b^	1,927	0	601	249	6
Lumbar punctures (includes “retest” ^c^)	1,806	0	350	204	8
RBANS^d^ tests	2,014	1,561	1,180	396	90
CDR^e^ tests	2,024	1,556	1,181	394	90

a Blood sample to measure *APOE* is only collected at baseline visit as per protocol.

b MRI scan is not performed at 6-month visit as per protocol.

c Lumbar puncture is not performed at 6-month visit as per protocol.

d Repeatable Battery for the Assessment of Neuropsychological Status.

e Clinical Dementia Rating Scale (blind rater).

All data, images, and samples from the EPAD LCS have been released as V.IMI (V = version) and are now freely available to all researchers globally *via* the Alzheimer's Disease Data Initiative's (ADDI) online platform, the AD Workbench (https://www.alzheimersdata.org/ad-workbench). The AD Workbench was publicly launched in November 2020 after a successful pilot that was supported by a coalition of organizations and industry partners interested in improving Alzheimer's and related dementia data sharing (https://www.alzheimersdata.org/about-addi). The EPAD dataset was the first full dataset to be made available on the AD Workbench and is the most highly requested dataset having received over 200 data access requests to date.

**Figure 2:**
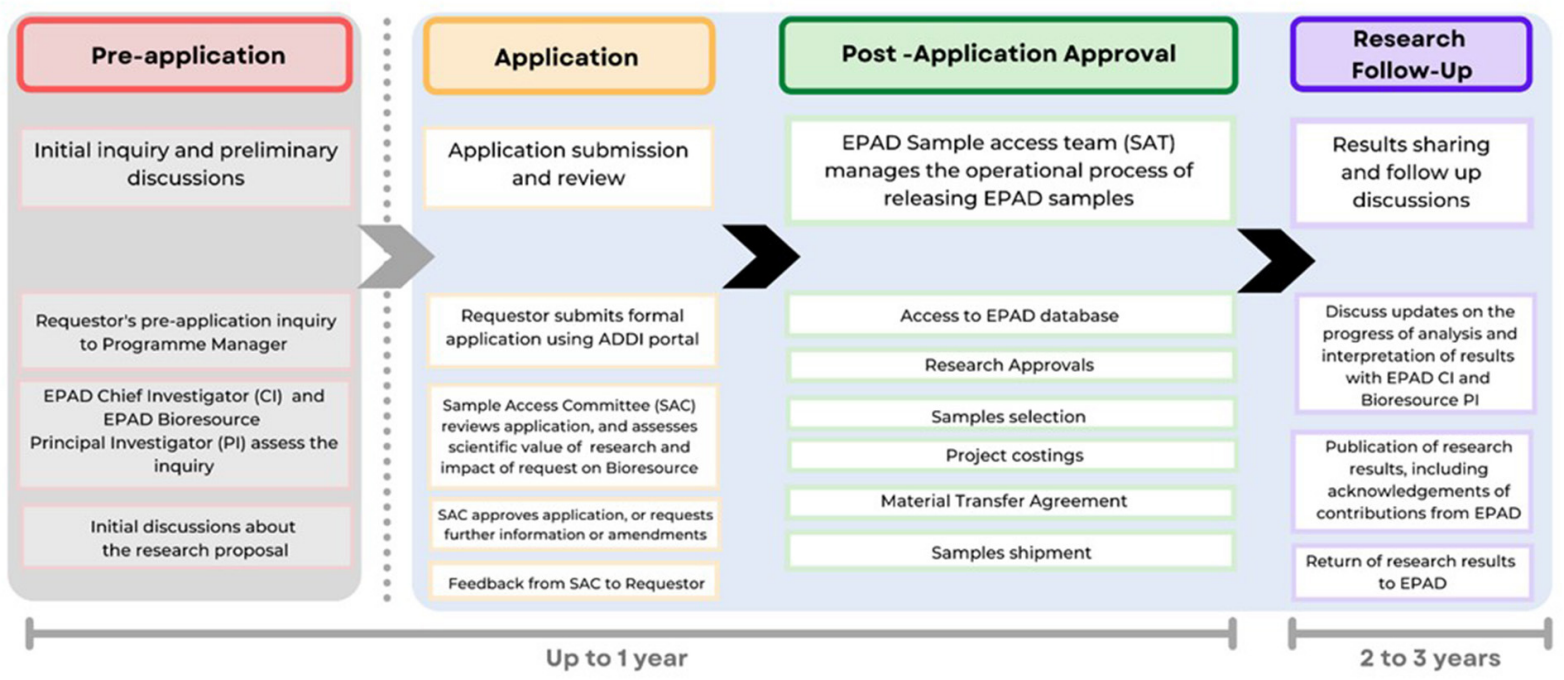
Requestor's journey to access EPAD samples and corresponding data.

The EPAD LCS created a huge biobank of more than 100,000 samples (CSF, blood, saliva, and urine), all stored in a single location at the Roslin Institute within the University of Edinburgh under optimal conditions. EPAD works on the principle that samples should be used and not stored indefinitely for (potential) future use and also that access should not be prohibited by costly access requirements. In essence, access should only be affected by the quality of the scientific question and the willingness to share derived data back into the main EPAD database. Data access applications are processed within several business days of the request and image requests are processed in 4 weeks. For sample access, researchers are encouraged to make informal inquiries about the scope of their research and to discuss the range of samples EPAD can offer. Sample access is governed by the rules of the Sample Access Committee that were designed with reference to the terms of the IMI-EPAD project agreement. The sample access process is illustrated in Figure 2 and can be started at https://ep-ad.org/samples-access/.

### Description of dataset and study methodology

As the EPAD project progressed, four datasets were made freely available ensuring the use of the data for the AD research community worldwide:

1. EPAD LCS V500.0, which includes data from the first 500 people to enter the cohort;
2. EPAD LCS V1500.0, which includes data from the first 1,500 people to enter the cohort;
3. EPAD LCS V500.1, which includes updated data from the first 500 participants, including 1-year follow-up data; and
4. EPAD LCS Version.IMI (V.IMI), which includes the final longitudinal data with cognitive, clinical, biomarker, and neuroimaging and lifestyle risk factor datasets from the over 2,000 participants of the EPAD LCS.

Each dataset was registered to a DOI for unique and specific identification of the dataset in publications and reference materials. To learn what data and associated metadata are available in the EPAD data release, visit the EPAD website (https://ep-ad.org/).

For a detailed overview of the study methodology and outcomes, refer to the V500.0 baseline data release article (9). The V500.0 is also the dataset used in many of the analyses described in the following section summarizing key findings to date.

To access all the data collected and processed during the IMI period of EPAD, please request the latest and final EPAD dataset (V.IMI) on the AD Workbench.

### Summary of key findings to date

At the time of writing this article, there are 42 EPAD-associated articles, spanning a broad range of topics; 17 results articles, nine review articles, seven methods articles, six results articles funded or associated with EPAD but not using EPAD LCS data, two editorials, and one article on data access. The articles have included 204 authors, across 94 institutions in 16 different countries. The 17 results articles include 115 individual authors from 63 institutions in 14 countries. Except for one article, all authors are from Europe or the USA. Authors were affiliated with academic institutions, charity organizations, SMEs, and pharmaceutical companies, demonstrating the public-private partnership continued from the set up and running of the project through to dissemination. Table 3 gives an overview of the results articles and findings; all articles are also listed on the EPAD website (www.ep-ad.org).

**Table 3:** Overview of results papers originating from analysis of EPAD data or EPAD participants.

**Publication title**	**Theme**	**Summary of main findings**
Application of the ATN classification scheme in a population without dementia: Findings from the EPAD cohort (10)	Biomarkers	• Used the ATN framework to define participants by biomarker status • 57.1% of participant were A-T-N- • 32.5% of participants were on the AD continuum • 10.4% of participant were suspected non-Alzheimer's pathology • Age and cerebrovascular burden increased with biomarker positivity • Cognitive dysfunction appeared with phosphorylated tau positivity (T+)
Associations between multimorbidity and cerebrospinal fluid amyloid: a cross-sectional analysis of the European Prevention of Alzheimer's Dementia (EPAD) V500.0 cohort (11)	Biomarkers	• Analyzed for associations between multimorbidity and cerebrospinal fluid (CSF) amyloid • Each additional condition was associated with a decreased likelihood of amyloid positivity (when using <1000pg/ml as cut off) • Each additional condition was associated with an increase in CSF amyloid of 54.2 pg/ml (95% CI: 9.9–98.5) • Having two or more conditions was inversely associated with amyloid positivity compared to one or no conditions
Cognitive Dispersion is not associated with cerebrospinal fluid biomarkers of Alzheimer's disease: results from the European Prevention of Alzheimer's Dementia (EPAD) v500.0 cohort (12)	Cognition and biomarkers	• Analyzed for associations between cognitive dispersion and CSF biomarkers • Found no significant associations between cognitive dispersions and any of the CSF analytes or categorical amyloid positivity • Greater cognitive dispersion seen in participants who were older and those who had less education
Cognitive functions as predictors of Alzheimer's disease biomarker status in the European Prevention of Alzheimer's Dementia cohort (13)	Cognition and biomarkers	• Analyzed for predictive value of cognitive functions for Alzheimer's disease biomarker status • Tau was significantly associated with an episodic verbal memory task • Amyloid beta was significantly associated with a central executive task
Cross-sectional associations between sleep quality reports and core Alzheimer's disease biomarkers in cognitively unimpaired adults from the European Prevention of Alzheimer's Dementia Longitudinal Cohort Study (EPAD LCS) (14)	Sleep and biomarkers	• Analyzed for associations (cross-sectionally and longitudinally) between self-reported sleep and CSF AD biomarkers • Cross-sectional analysis found that poor sleep quality was associated with higher CSF tTau, shorter sleep duration was associated with higher CSF pTau and tTau • Greater sleep disturbance was associated with lower CSF Aβ both cross-sectionally and longitudinally
Disease modeling of cognitive outcomes and biomarkers in the European Prevention of Alzheimer's Dementia longitudinal cohort (15)	Disease modeling	• Developed a two-stage approach for modeling of longitudinal cognitive and clinical outcomes • Demonstrated clinical and biological utility in incorporating multiple factors to modeling trajectory, subgroup identification and predictive power
European Prevention of Alzheimer's Dementia Registry: Recruitment and prescreening approach for a longitudinal cohort and prevention trials (16)	Recruitment methods	• Analysis of feasibility of recruitment approach employed in EPAD LCS • Demonstrated success of using a virtual registry to preselect participants for AD studies
Involving research participants in a pan-European research initiative: the EPAD participant panel experience (17)	Participant involvement	• Analysis of the impact of the participant involvement panels • Panel members provided important and useful feedback on study documentation • Panel members involved with design of new study materials • Panel members represented the project at national and international meetings
Interactions between apolipoprotein E, sex, and amyloid-beta on cerebrospinal fluid p-tau levels in the European prevention of Alzheimer's dementia longitudinal cohort study (EPAD LCS) (18)	Biomarkers	• Analyzed for associations between CSF amyloid and p-Tau by sex and *APOE ε4* carrier status • There was a significant interaction between sex, *APOE ε4* and amyloid-beta on pTau • This interaction appeared to be significant in male but not female participants • In female participants, those who were *APOE ε4* carriers with higher CSF amyloid had significantly elevated pTau levels.
Lived time and the affordances of clinical research participation (19)	Participant involvement	• Analysis of interviews with study participants to understand their experiences of involvement • Taking part in research gave a role, an opportunity to keep busy and stay useful • Incidental benefit of receiving a full health check up, an ‘MOT' • Future research participant in clinical trials largely approach through an altruistic lens
Prediction of Alzheimer's disease biomarker status defined by the “ATN framework” among cognitively healthy individuals: results from the EPAD longitudinal cohort study (20)	Biomarkers	• Used the ATN framework to define participants by biomarker status • Key variables differed between ATN biomarker groups: age, *APOE ε4*, family history, body mass index, mini mental state examination score and white matter lesions • Prediction of AD pathology improved by adding these key variables to model • Addition of established risk composite scores did not improve predictive power
Prescreening for European Prevention of Alzheimer's Dementia (EPAD) trial-ready cohort: impact of AD risk factors and recruitment settings (6)	Recruitment methods	• Analysis of the impact of risk factors and recruitment settings on prescreening • Participation in the EPAD LCS was associated with lower age, higher education, male sex and family history of dementia • Amyloid positivity was associated with higher age and *APOE ε4* allele carrier status • Results were similar across all prescreen settings (clinical cohort, research in-person cohort, research online cohort, population based cohort)
Regional associations of white matter hyperintensities and early cortical amyloid pathology (21)	Imaging	• Component analysis of white matter hyperintensity (WMH) patterns • Component 1: fronto-pariteal WMH pattern association with amyloid in the medial orbitofrontal-precuneus, vascular risk and age; associated with lower global cognitive performance • Component 2: poster WMH pattern associated with amyloid in the precuneus-cuneus, less related to age and vascular risk; associated with lower memory scores
The European Prevention of Alzheimer's Dementia (EPAD) Longitudinal Cohort Study: Baseline Data Release V500.0 (9)	Baseline data release	• Description of the first 500 participants baselined into the EPAD LCS • Mean age of cohort 66.4 (6.7) years, 47.8% male • Participants represented a spectrum of normal aging (CDR=0, Amyloid -), preclinical AD (CDR=0, Amyloid +), prodromal AD (CDR=0.5, Amyloid +), and non-AD related cognitive change (CDR=0.5, Amyloid-)
The influence of diversity on the measurement of functional impairment: An international validation of the Amsterdam IADL Questionnaire in eight countries (22)	Functional assessment validation	• Cross-cultural validation of the functional assessment questionnaire • Limited bias evident for age, gender, education, and culture in the measurement of functional impairment
“Ready for What” Timing and Speculation in Alzheimer's Disease Drug Development (23)	Conceptualization of readiness	• Analysis of interviews with EPAD associated staff on meaning of readiness • Discussion of importance of temporal specificity regarding the concept of readiness in preclinical research • Trial readiness is a challenging concept to grasp within a field with a highly speculate drug development pipeline
Self-reported diabetes is associated with allocentric spatial processing in the European Prevention of Alzheimer's Dementia Longitudinal Cohort Study (17)	Cognition	• Analysis of associations between self-reported diabetes and allocentric spatial processing test performance • Significantly poorer performance on the Four Mountain Test for those with diabetes compared to those without, with a global pattern of cognitive impairment • Poorer performance on the Four Mountains Test and attention index of the Repeatable Battery for the Assessment of Neuropsychological Status was specific to diabetes, compared to obesity and hypertension
Assessing and disclosing test results for “mild cognitive impairment”: the perspective of old age psychiatrists in Scotland (24)	Associated results paper	• Analysis of clinicians interviews on the topic of MCI diagnostic disclosure • Lack of specific and sensitivity assessment measures for identifying etiology of MCI available in clinical practice • Direct impact on management on individuals with MCI

#### Biomarkers

Nine published articles have reported on biomarkers, imaging, and cognition. The Amyloid/Tau/Neurodegeneration (ATN) framework has been used by two articles to define participants by biomarker status. Through this framework, 57.1% of the EPAD LCS cohort included by Ingala et al. (10) were A-T-N-, 32.5% were on the AD-continuum, and 10.4% suspected non-Alzheimer's pathology. The authors found that both age and cerebrovascular burden progressed with biomarker positivity. Additionally, phosphorylated tau was associated with cognitive dysfunction in individuals without dementia, and memory and language domains were affected in the earliest stages of neurodegeneration across the cohort (10).

Calvin et al. (20) found significant differences by age, *APOE* ε*4*, family history, body mass index, mini-mental state examination, and white matter lesion (WML) volume across the ATN groups. Prediction of AD pathology improved by adding these components to a ROC curve; however, there was no additional value in including established dementia composite risk scores (20).

A further study considering disease modeling applied a two-stage approach utilizing longitudinal cognitive and clinical outcomes, biomarkers (baseline and longitudinal), and risk factor data. The two-stage approach demonstrated clinical and biological utilities in trajectory stratification and was able to identify subgroups of interest in the dataset (15).

AD biomarkers, specifically cerebrospinal fluid (CSF) Aβ1-42, have also been investigated about multimorbidity in the EPAD cohort. When including the number of conditions as a continuous variable representing multimorbidity, each additional condition was associated with a decreased likelihood of amyloid positivity and higher CSF Aβ concentrations, suggesting that the established association between multimorbidity and dementia may be due to a pathway other than amyloid (11).

An analysis of sex differences with regard to *APOE* ε*4* carrier status found a significant interaction of sex, *APOE* ε*4*, and Aβ, with male participants showing a stronger association between *APOE* ε*4* and Aβ on pTau compared to female participants. In this same study, female *APOE* ε*4* carriers, but not male, with high levels of CSF Aβ had significantly elevated pTau compared to non-carriers, suggesting that accumulation of pTau may be independent of amyloid for women (18).

Both cross-sectional and longitudinal analyses of the EPAD dataset found associations between self-reported measures of sleep and AD biomarkers. Sleep disturbance was associated with lower CSF Aβ concentrations at both baseline and longitudinal follow-up, poor sleep quality was associated with higher CSF tTau at baseline and short sleep duration was associated with higher CSF pTau and tTau (14).

Analysis of cognitive results has also given rise to interesting findings. One study investigating the concept of cognitive dispersion found that it was associated with both age and education, but not with AD pathology, in the EPAD cohort (12). A second study investigating associations between Aβ, tau, and specific cognitive tests identified biomarker-specific profiles of cognitive impairment. A primarily hippocampal task was associated with higher levels of tau, while a frontal executive task was associated with higher levels of Aβ (13).

Focusing on neuroimaging, Lorenzini et al. (21) investigated associations among amyloid, age, and vascular risk with white matter hyperintensities (WMH). The analysis found a two-component pattern, whereby the first component identified a frontoparietal WMH pattern which was associated with amyloid (in the medial orbitofrontal precuneus), vascular risk, and age, and, in turn, was associated with lower performance in all cognitive domains; and the second component with a posterior WMH pattern associated primarily with precuneus-cuneus amyloid and poorer performance in tasks of memory (21).

Furthermore, the IMI-funded Amyloid Imaging to Prevent Alzheimer's Disease (AMYPAD) study was a sister project to the EPAD study and focused entirely on amyloid as one of the hallmark biomarkers in the AD process. The study was designed as two distinct projects: the Prognostic Natural History Study (PNHS) which performed amyloid-PET in the EPAD LCS cohort as well as later on other similar cohorts to investigate the added value of amyloid imaging in early detection of AD (25) and the Diagnostic and Patient Management Study (DPMS) aimed to assess the clinical impact and cost-effectiveness of amyloid-PET in memory clinic patients (26). The close collaboration with the AMYPAD project illustrates how data from EPAD benefits AD projects more broadly.

These articles on biomarker discoveries highlight the deep phenotyping available in the EPAD LCS and demonstrate some important emerging findings, particularly around the effects on cognition of tau in participants without dementia and the need to expand beyond amyloid when considering the multifactorial risk factors for AD.

#### Cognition

One article led by Gregory et al. (27) tested for associations between cardiovascular health and cognitive test performance, finding associations between having diabetes and performing significantly more poorly on the Four Mountains Test (FMT), a test of allocentric processing. This was on the background of a global cognitive impairment seen for those participants with self-reported diabetes, as measured using the Repeatable Battery for the Assessment of Neuropsychological Status (RBANS). Analysis of associations between cognitive test performance and both obesity and hypertension found patterns of impairment, however, neither was as global as diabetes, and only the FMT was specific to those with diabetes, suggesting that this may be an important task to identify early cognitive impairment in a high risk for future dementia group (27).

#### Participant involvement

Two articles presented data on participant involvement, one from analysis of reasons why participants joined a cohort and platform trial, and the second focused on panel achievements. Analysis of interviews with older adults in a clinical trial platform found that participants spoke about being involved in research giving them a role, keeping busy, staying useful, as well as receiving the incidental benefit of getting a full health checkup, while there was mainly an altruistic motivation when considering possible future clinical trial participation (19). The findings suggest that participants may not expect to personally benefit from future clinical trials but wish to contribute toward drug development in AD, thus making them part of a future in which preventative medicine could 1 day help them, or people like them. The participant panel structure within EPAD was found to have a wide impact on the overall project, with examples of benefits including feedback on documentation, support on the design of novel recruitment materials, and representation of EPAD at national and international meetings (17). These articles evidence the important role participants played, both as data and sample donors, and active stakeholders in the EPAD project, lending credence to the value of this dataset to the wider AD community.

#### Recruitment methods

Given the novel recruitment methods used in the EPAD project, two results articles exclusively reported on this. The first reviewed the set up and utility of the virtual registry and found that such a system can be used for the preselection of participants for AD studies (16). The second article reviewed participation rates and found that compared to those who declined participation, those enrolling in the EPAD LCS were younger, more educated, more likely to be male, and have a family history of dementia (6). This evidence can inform future cohort and trial recruitment strategies and is also useful to set the context for who the participants included in the EPAD LCS are.

#### Other

Other articles include a conceptualization of what “readiness” means (23) and cross-cultural validation of the Amsterdam Instrumental Activities of Daily Living (IADL) (22). Articles funded by EPAD have explored topics, such as clinicians' experience of MCI disclosure, with evidence demonstrating a lack of specific and sensitive assessment methods for identifying the etiology of MCI in clinical practice, which may impact the management of individuals with MCI (24).

The broad scope of articles affiliated with EPAD shows the multi-disciplinary approach that was taken in the work package set up, with continuing diverse academic collaborations as a key legacy of this project.

### EPAD early career researcher support

Supporting early career researchers (ECRs) was a core principle from the outset of EPAD and was primarily achieved through the establishment of the EPAD Academy, and also through supporting Ph.D. research. The Academy, which was open for all ECRs affiliated with the EPAD project, aimed to identify and support junior researchers' needs for career advancement through specific activities, such as contributions to scientific publications, participation in conferences, and development of guidelines and follow-on studies. The academy activities, involving nearly 100 ECRs, included a webinar series, workshops at the General Assembly, and hosting of ECRs at partner organizations. Ultimately, the academy helped to nurture the next generation of AD researchers and thought leaders by creating and facilitating opportunities for junior researchers' career advancement, with many of the EPAD Academy members leading and co-authoring the publications arising from EPAD. This ECR support has continued through the IMI-funded Neuronet's annual events held for ECRs working within the IMI neurodegenerative disease portfolio. The EPAD leadership recognizes that continuing the networking opportunities are critical to our next generation of scientists.

### EPAD impact on the patient community

EPAD has left a tangible clinical legacy with there being no doubt that the community of clinicians, academics, and research participants underpinned the European “Brain Health” direction. This movement began with considering how best to prepare for future needs with the potential arrival of disease-modifying therapies (28, 29). Rapidly, these discussions have started to translate to new clinical care pathways, with the exemplary models of Brain Health Scotland (30) and the Davos Alzheimer's Collaborative (DAC) Health Care Readiness Flagship (31). The European Taskforce for Brain Health Services has released a series of manuals detailing the set up (32–37) with many recommendations reflecting the EPAD protocol.

### EPAD impact on the pharmaceutical industry and SMEs

Although the PoC did not open to recruitment, EPAD nevertheless had an important impact on the AD pharmaceutical industry. First, the cohort continues to exist at local sites, with most participants having consented to re-contact and with local follow-up studies underway at some sites. This allows accessing a well-phenotyped pool of trial-ready participants, to de-risk clinical programs, as well as a network of highly trained sites keen to engage in preclinical AD clinical research studies. The set up of EPAD also optimized adaptive design methodologies through modeling and simulation efforts, as well as recruitment tactics and patient outreach. The process of establishing EPAD also developed a deep understanding of both public and private organizations of the European Union ecosystem, affording networking opportunities across the consortium and informal interactions with authorities and regulators, as well as key opinion leader organizations. This community building was an integral part of the public-private partnership. The community that was built within EPAD between all types of partners led to a breakdown of the traditional silos of academic and healthcare vs. industry. In particular, participant panel groups were afforded opportunities to meet staff employed in the private sector, allowing both parties to learn more about the research environment from novel perspectives. While not run, the PoC drug-ready platform infrastructure exists and could be re-opened or replicated to benefit from the existing protocol, legal framework, vendor agreements, and regulatory acceptability work. The ongoing opportunity to access data, and importantly biological samples, continues to be important to the pharmaceutical industry and many SMEs to inform ongoing clinical development programs. Several SMEs also benefitted from their involvement, winning additional contracts for future aligned work.

More difficult to capture is the community that was built within EPAD between all types of partners, breaking down the traditional silos of academic and healthcare vs. industry.

## Discussion

The EPAD project received €64 million in financial investment, recruited over 2,000 research participants into the LCS, and involved more than 400 researchers across 39 partner organizations. The ongoing EPAD LCS data and biobank access are key outcomes of this work, with both freely available and easily accessible *via* ADDI's AD Workbench platform and the Sample Access Committee. A growing number of EPAD-associated publications demonstrates the unique value of this cohort, with results to date suggesting many interesting future research avenues to explore. It is expected that in the coming years, data analysis from numerous research groups will yield many important observations to be published and therein influence our collective knowledge of many biological and clinical aspects of AD. Moreover, further follow-up of research participants who were in the EPAD LCS will continue at both local and national levels under separate protocols and data can be linked back to the IMI data as well as across the new follow-up projects through designed-in data interoperability using, for example, ADDI's AD Workbench. Other legacies of EPAD include benefits to ECR careers, the trained and experienced established site network, and lasting impacts to industry partners.

Securing ongoing funding for EPAD was seriously hampered by the 2020/21 COVID-19 pandemic which curtailed the ability to set up new clinical trials or continue to follow-up with EPAD research participants. Platform trials helped defeat COVID-19; from 2021 onward, they will also be key to defeating one of the greater challenges of our time which is AD. The framework established for the EPAD PoC will undoubtedly be a critical learning opportunity for these future platform trials (38), alongside learnings from ongoing platform trials developed within the Dominantly Inherited Alzheimer's Network Trials Unit (DIAN-TU) (39).

There were many clear strengths of EPAD. First, the data collected were of the highest quality. Although the cohort itself was not a drug study, as data collected from the LCS would potentially be used as run-in data in a future clinical trial, the LCS data were collected in accordance with Good Clinical Practice (GCP) and Clinical Data Interchange Standards Consortium (C-DISC) standards/guidelines. This is unusual for an observational study and involved high levels of quality control checks, meaning the data are robust and reliable. Being able to trust in the validity of data collected by someone else is of the utmost importance to researchers accessing datasets. The EPAD LCS also forms the largest collection of imaging and CSF data in preclinical AD globally, offering both cross-sectional baseline data and longitudinal follow-up. With some centers already working on local follow-up studies, this longitudinal information collection is ongoing and will provide important opportunities to answer some of our key research questions in the field of AD. The centering of participants' involvement was also seen as key to EPAD from its initiation and has been identified both internally and externally as a strength of EPAD. The panel involvement from multiple centers and countries allowed the project to collect data that were not only meaningful to academic and industry partners but also those living with the greatest risk of future AD. Delivering research that is important to those facing the greatest burden of this disease must be at the heart of what we in the AD research community do. There was also, despite challenges, the achievement of redirecting science and operational elements to build this novel approach to tackling AD through secondary prevention. The community within EPAD was largely responsible for this, through engaging actively in supporting the approach to fostering junior talent through the EPAD academy.

There were also several limitations to EPAD. The main challenge in the EPAD LCS was enrolling individuals in a cohort who were also eligible for clinical trial opportunities. This was particularly keenly noticed for individuals with MCI, who were understandably eager to join drug trials rather than a cohort. This resulted in some of the participants with MCI dropping out of the LCS prematurely. In addition, the cohort should be acknowledged as underrepresenting certain parts of the European population with an overrepresentation of white and highly educated participants. Although this is true of most cohort studies in this area, future cohorts should endeavor to build more inclusive recruitment mechanisms. Local follow-up studies are working to redress this balance, with the EPAD Scotland study as an example where new participants without tertiary education are being recruited to better reflect the general Scottish population. Despite the positive engagement in challenging the status quo, it remained difficult to secure the willingness of numerous third-party organiztions or departments within partner organizations to innovate in legal, research governance, and institutional cultural change. More specific to the PoC, although intervention owners were enthusiastic about using a platform trial to run PoC studies in AD, they were ultimately reluctant to hand over the sponsorship for a critical asset to a university. This, in combination with the difficulties in agreeing on the common legal framework that was usable and acceptable across stakeholders were the main contributors to the PoC trials not starting, and needs to be addressed in future efforts in this area.

To conclude the EPAD project, it has been a great example of what public and private partnerships can achieve and IMI funding was critical to this. ADDI, GAP, IMI-Neuronet, and follow-on funding from the Alzheimer's Association for the data and sample access systems ensure that the EPAD assets will be maintained and, as and when sponsors seek a new platform trial to be established, the learnings from EPAD will ensure that this can be developed to be even more successful than this first pan-European attempt.

## Data availability statement

The original contributions presented in the study are included in the article/supplementary material, further inquiries can be directed to the corresponding author.

## Ethics statement

The studies involving human participants were reviewed and approved by NHS Research Ethics Committee. The patients/participants provided their written informed consent to participate in this study.

## Author contributions

SS and SGr: conceptualization, writing—original draft, and writing—reviewing and editing. MC and CB: writing—reviewing and editing. SGe and CR: conceptualization and writing-reviewing and editing. All authors contributed to the article and approved the submitted version.

## Funding

This work used data from the EPAD project which received support from the EU/EFPIA Innovative Medicines Initiative Joint Undertaking EPAD grant agreement no 115736 and an Alzheimer's Association Grant (SG-21-8180990-EPAD).

## Conflict of interest

Author MC was employed by Alzheimer's Disease Data Initiative. Author CB was employed by Alzheimer Europe. Author SGe was employed by Janssen Research and Development, a Division of Janssen Pharmaceutica NV. Author CR was employed by Brain Health Scotland. The remaining authors declare that the research was conducted in the absence of any commercial or financial relationships that could be construed as a potential conflict of interest.

## Publisher's note

All claims expressed in this article are solely those of the authors and do not necessarily represent those of their affiliated organizations, or those of the publisher, the editors and the reviewers. Any product that may be evaluated in this article, or claim that may be made by its manufacturer, is not guaranteed or endorsed by the publisher.
